# QM/MM Well-Tempered Metadynamics Study of the Mechanism of XBP1 mRNA Cleavage by Inositol Requiring Enzyme 1α RNase

**DOI:** 10.1021/acs.jcim.2c00735

**Published:** 2022-08-12

**Authors:** Sayyed Jalil Mahdizadeh, Emil Pålsson, Antonio Carlesso, Eric Chevet, Leif A. Eriksson

**Affiliations:** † Department of Chemistry and Molecular Biology, University of Gothenburg, 405 30 Göteborg, Sweden; ‡ Faculty of Biomedical Sciences, Euler Institute, Università della Svizzera Italiana (USI),, Lugano 6904, Switzerland; § INSERM U1242, University of Rennes 1, 35000 Rennes, France; ∥ Centre Eugène Marquis, 35000 Rennes, France; ⊥ Department of Pharmacology, Sahlgrenska Academy, University of Gothenburg, 405 30 Göteborg, Sweden

## Abstract

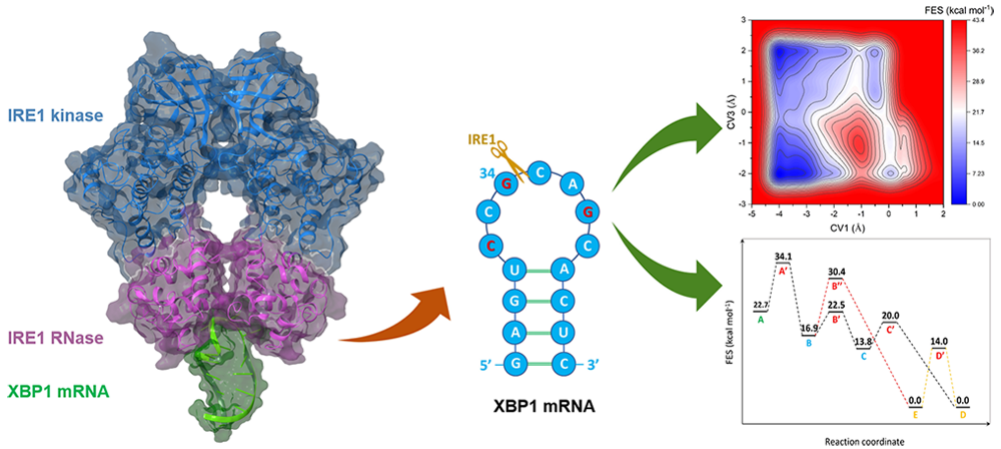

A range of *in silico* methodologies were herein employed to study the unconventional XBP1 mRNA cleavage mechanism performed by the unfolded protein response (UPR) mediator Inositol Requiring Enzyme 1α (IRE1). Using Protein–RNA molecular docking along with a series of extensive restrained/unrestrained atomistic molecular dynamics (MD) simulations, the dynamical behavior of the system was evaluated and a reliable model of the IRE1/XBP1 mRNA complex was constructed. From a series of well-converged quantum mechanics molecular mechanics well-tempered metadynamics (QM/MM WT-MetaD) simulations using the Grimme dispersion interaction corrected semiempirical parametrization method 6 level of theory (PM6-D3) and the AMBER14SB-OL3 force field, the free energy profile of the cleavage mechanism was determined, along with intermediates and transition state structures. The results show two distinct reaction paths based on general acid–general base type mechanisms, with different activation energies that perfectly match observations from experimental mutagenesis data. The study brings unique atomistic insights into the cleavage mechanism of XBP1 mRNA by IRE1 and clarifies the roles of the catalytic residues H910 and Y892. Increased understanding of the details in UPR signaling can assist in the development of new therapeutic agents for its modulation.

## Introduction

The endoplasmic reticulum (ER) is an organelle present in all eukaryotic cells that is involved in maintaining cellular homeostasis.^1^ Nascent protein chains enter the ER where they are post-translationally modified and folded by ER-resident foldases, chaperones, and quality control components. Once their correct conformation is acquired, mature proteins exit the ER and disseminate throughout the cell to achieve their functions. Protein folding in the ER can be challenged when the folding demand exceeds the ER folding capacity, in turn leading to the accumulation of improperly folded proteins in this compartment. This situation is known as ER stress. To cope with ER stress and restore cellular homeostasis, an adaptive response named the “unfolded protein response” (UPR) is triggered. The UPR monitors and regulates protein folding within the ER by temporarily increasing the protein folding efficacy to attenuate the accumulation of unfolded/misfolded proteins and increase ER-associated degradation of terminally misfolded proteins.^2^ In metazoans, the UPR is mediated by three main sensors: Inositol Requiring Enzyme 1α (hereafter referred to as IRE1), Protein kinase R-like Endoplasmic Reticulum Kinase (PERK), and Activating Transcription Factor 6α (ATF6α).^1^

**Figure 1:**
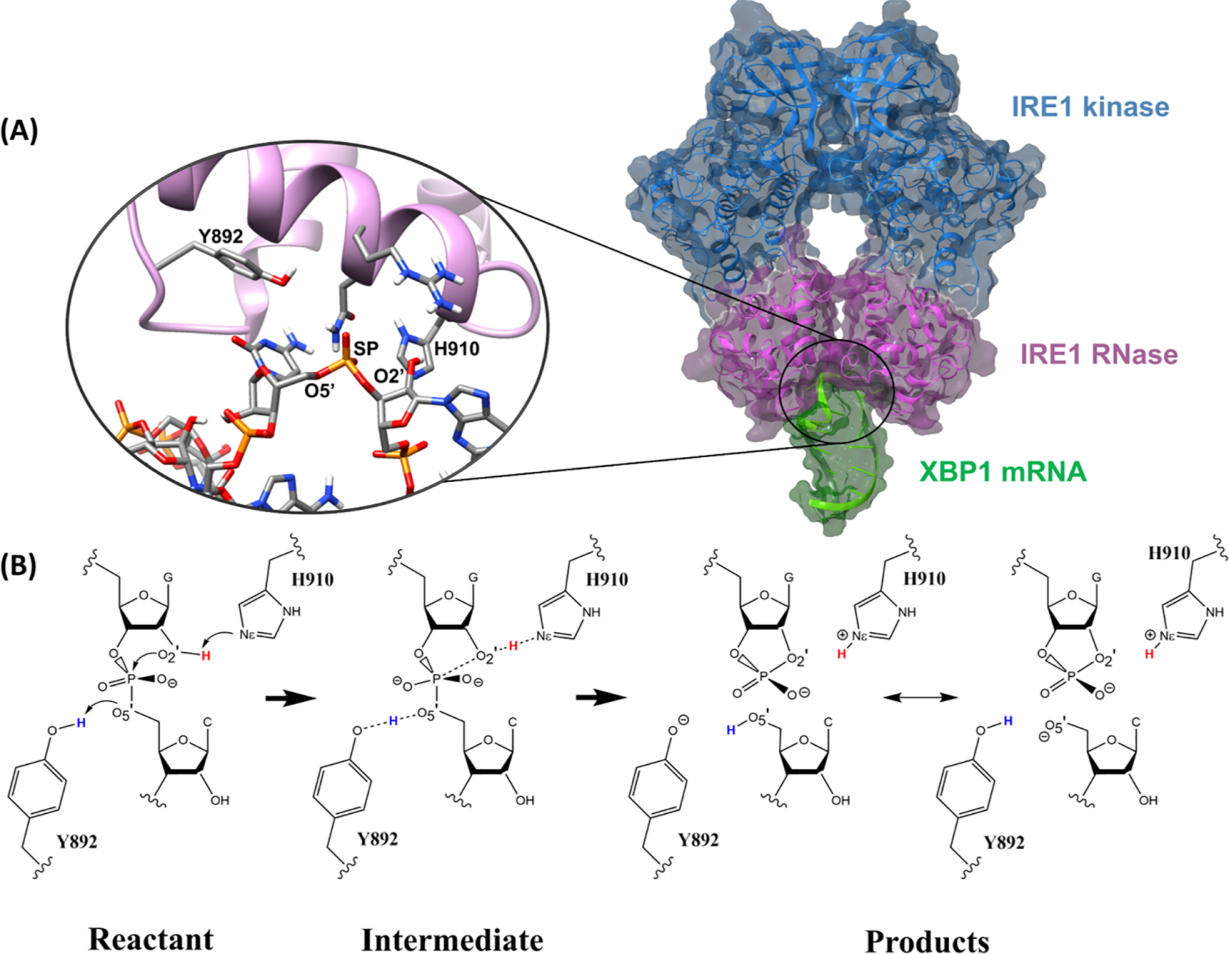
(A) *h*IRE1 back-to-back dimer in complex with mRNA XBP1 single stem-loop complex generated from molecular docking calculations. The kinase and RNase domains of IRE1 dimer are shown in blue and pink, respectively. The luminal and transmembrane domains of *h*IRE1 are not shown in the figure. XBP1 mRNA single stem-loop is represented in green. The zoomed-in picture illustrates the catalytic residues (H910 and Y892) within the active site of the *h*IRE1 RNase in which the XBP1 mRNA cleavage reaction takes place. (B) Schematic 2D illustration of a concerted general acid–general base (GA–GB) reaction mechanism. The catalytic Histidine (H910) acts as a GB and initiates the reaction by abstracting a proton (red) from the Guanosine O2′ atom. Tyrosine (Y892) acts as a GA by donating its phenolic proton (blue) to the Cytidine O5′ atom.

IRE1 is a bifunctional type-I ER-resident transmembrane protein,^2^ composed an ER luminal “sensor” domain, a single pass membrane traversing domain, and a cytosolic part containing both kinase and RNase domains. Upon accumulation of unfolded/misfolded proteins, IRE1 activation is triggered^3,4^ (the details of which are still under debate),^5−7^ whereby the RNase domain located at the down-end part of the interface between two IRE1 monomers (Figure 1A) excises a 26-nucleotide intron from the X-box binding protein 1 (XBP1) mRNA.^8^ XBP1 mRNA cleavage by IRE1 RNase is specific through the cleavage of select stem-loops.^9,10^ Furthermore, for XBP1 mRNA splicing to take place, IRE1 tetramers (pairs of dimers) are required because spatial constraints of the RNase binding pocket inhibit the concurrent binding of two XBP1 mRNA stem-loops.^4^ After intron excision, the two resulting exon ends are ligated by the tRNA ligase RtcB, and the transformed XBP1s mRNA (“s” for spliced) is translated into a potent transcriptional activator that controls the expression of UPR target genes.

The atomistic details underlying the IRE1 RNase-mediated XBP1 mRNA cleavage mechanism is not fully understood. However, a study on structural and functional bases for RNA cleavage by the *yeast* IRE1^4^ suggests a stepwise general acid–general base (GA–GB) mRNA cleavage mechanism where H1061 and Y1043 (H910 and Y892 in *human* IRE1, respectively) function as the GB and GA, respectively. The stepwise mechanism can be summarized as follows (Figure 1B): (a) The GB abstracts a proton from the O2′ position of a nucleotide and becomes positively charged. (b) The negatively charged O2′ performs a nucleophilic attack on the phosphorus atom of the scissile phosphate, leading to the formation of a pentavalent dianionic phosphate intermediate. (c) The O5′ atom of the leaving nucleotide abstracts a proton from the GA leading to a complete cleavage of the mRNA sequence and formation of a 2′-3′ cyclic phosphate. R1056 and N1057 (R905 and N906 in *h*IRE1) also play important roles in this mechanism by coordination of the scissile phosphate and charge stabilization. The same GA–GB mechanism was also proposed for other ribonuclease enzymes such as ribonucleases T1 and A.^11^ The stepwise GA–GB mechanism is considered to be favored over the classical concerted mechanism^11^ as it allows for the protonated, positively charged GB to electrostatically interact with and stabilize the pentavalent dianionic phosphate intermediate. Mutagenesis analysis in *y*IRE1^4^ showed that GB (the catalytic histidine) is essential for the cleavage mechanism since H1061N mutation displays a >300,000-fold rate reduction. It has also been shown that mutation of the catalytic histidine to alanine, asparagine, or glutamine diminishes catalytic activity of fungal ribonuclease T1 and mammalian RNase A by 1000- to 10,000-fold.^12−14^ In addition, mutation of the GA Tyrosine in *y*IRE1 to phenylalanine (Y1043F) does not block the IRE1 RNase activity but reduces the reaction rate by ∼10-fold.^4^

In the absence of an atomic resolution cocrystal structure of the IRE1–RNA complex, molecular modeling approaches can be used to provide an approximate picture of the interactions and investigate the RNA cleavage mechanisms. Herein, multiscale *in silico* techniques (i.e., RNA modeling, molecular docking, molecular dynamics simulations, and quantum mechanics/molecular mechanics well-tempered metadynamics simulations) were employed to (a) construct IRE1 back-to-back dimer/XBP1 mRNA single stem-loop complexes as models which take the dynamics of the three-dimensional conformation into consideration (Figure 1A) and (b) quantitatively investigate the cleavage mechanism of mRNA XBP1 by the IRE1 RNase domain (Figure 1B). An IRE1 back-to-back dimer (receptor) and one of the single stem-loops of the mRNA XBP1 (ligand) were used to facilitate the calculations and reduce the complexity of the system.

## Methods

### IRE1 Back-to-Back Dimer

The *h*IRE1 back-to-back dimer (Figure 1A) was obtained from the RCSB Protein Data Bank (PDB ID, 4YZC;^8^ resolution, 2.49 Å). The crystal structure comprises a phosphorylated *h*IRE1 dimer complexed with a Staurosporine molecule in the kinase pocket of each monomer. The *h*IRE1 dimer is in its back-to-back conformation and has an activated cytosolic RNase domain. The dimer structure was prepared using the Schrodinger’s protein preparation wizard.^15^ The missing hydrogen atoms were added, and the Prime program^16,17^ was employed to model absent side chain atoms and missing loops. The protonation states of ionizable residues were determined using the PROPKA tool at pH 7.4. As a final refinement, the protein was subjected to a restrained minimization (RMSD = 0.3 Å) using the OPLS4 force field.^18^

### XBP1 mRNA Single Stem-Loop Modeling

**Figure 2:**
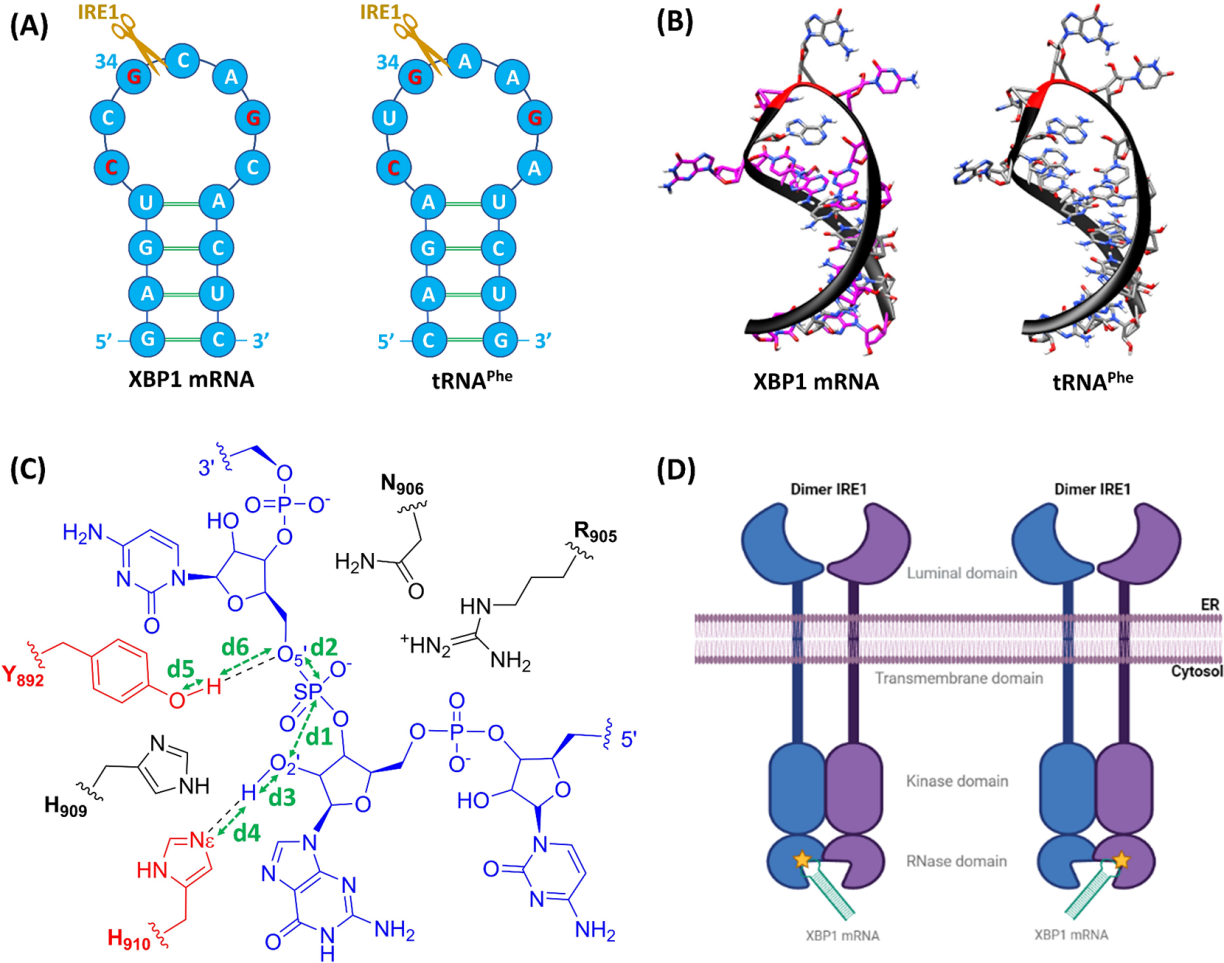
(A) 2D and (B) 3D structures of the modeled XBP1 mRNA single stem-loop and the template used (tRNA^Phe^) for the modeling. IRE1 can target and unconventionally cleave both RNA molecules as they share the same consensus sequence (5′-CNGNNGN-3′) within the stem-loops. The mutated residues in the XBP1 mRNA are shown in purple. (C) Composition of the QM subsystem (161 atoms) specified for the QM/MM WT-MetaD simulation. XBP1 stem-loop and two IRE1 catalytic residues (Y892 and H910) are shown in blue and red, respectively. The interatomic distances used to formulate the collective variables are shown by green arrows. (D) Two active catalytic centers (shown by asterisks) within the cleft between the RNase loops of the *h*IRE1 dimer.

The template (tRNA^Phe^) utilized in the construction of the mRNA XBP1 single stem-loop was received from the Protein Data Bank (PDB ID, 2ZM5;^19^ resolution, 2.55 Å). The crystal structure consists of the tRNA modification enzyme MiaA in complex with tRNA^Phe^. It has been shown^4^ that *y*IRE1 can recognize and cleave tRNA^Phe^. To model the XBP1 mRNA single stem-loop, a 15-nucleotide long sequence was cut out from the stem-loop of tRNA^Phe^. Thereafter, the bases within the stem-loop were mutated^20^ using the “swapna” tool in USCF Chimera^21^ to mimic the nucleotide sequence in one of the single stem-loops of *Homo sapiens* XBP1 mRNA (5′-GAGUCCGCAGCACUC-3′).^9^ Finally, the constructed XBP1 mRNA single stem-loop was prepared using the preparation wizard in the Schrodinger package^15^ (Figure 2A and B).

### Molecular Docking

Molecular docking was performed with HDOCK,^22^ a molecular docking web server specialized for protein–protein and protein–DNA/RNA interactions. The *h*IRE1 dimer was used as the receptor and the XBP1 mRNA single stem-loop as the ligand. It has been proven^4^ that the RNase domain of each monomer in the IRE1 dimer is catalytically active and can cleave XBP1 mRNA; i.e., there are two active catalytic centers within the cleft between the RNase loops of the IRE1 dimer (Figure 2D). Constraints were applied to guide the XBP1 mRNA single stem-loop toward the catalytic center of either monomers A or B. A successful molecular docking should be able to accommodate the XBP1 mRNA stem-loop in two equivalent orientations, one toward each monomer. The receptor constraints included the four catalytic residues Y892, R905, N906, and H910 from each *h*IRE1 monomer. The ligand constraints included the two nucleotides G34 and C35 which are connected by the scissile phosphate group.

### Molecular Dynamics Simulations

The best docking model from molecular docking calculations was subjected to a series of extensive MD simulations to further relax and refine the protein–RNA structure, evaluate the dynamical behavior of the system, achieve a better insight about the interatomic interactions, and extend the conformational sampling to find a plausible starting structure for the subsequent quantum mechanics/molecular mechanics well-tempered metadynamics (QM/MM WT-MetaD) simulations. All MD simulations were performed in the NPT ensemble using the Desmond MD simulation engine^23^ with an OPLS4 force field^18^ as implemented in the Schrodinger package. Two systems were explicitly solvated with a TIP3P water model^24^ under periodic boundary conditions in a cubic simulation box with a 15 Å buffer in each direction. The systems were neutralized by adding a proper number of K^+^ ions, and a 0.15 M MgCl_2_ salt concentration was considered. It has been shown that K^+^ ions play a role in the stabilization of RNA through phosphate backbones or via coordination to exocyclic groups on stacked nucleotides.^25^ MgCl_2_ was used because experimental evidence^26^ suggest that it results in a stability improvement of RNA molecules. The Nosé–Hoover^27^ thermostat (relaxation time of 1 ps) and Martyna–Tobias–Klein^28^ barostat (relaxation time of 2 ps) were applied during the simulations to set the systems temperature and pressure at 300 K and 1 atm, respectively. Long-range electrostatic energy and forces were calculated using the particle-mesh Ewald method.^29,30^ The SHAKE algorithm^31^ has been applied to all covalent bonds between hydrogen and heavy atoms, with a tolerance and maximum iterations of 10^–8^ and 8, respectively. The initial minimization and equilibration protocol comprised the following: (i) NVT Brownian dynamics with restraints on solute heavy atoms at *T* = 10 K for 100 ps, (ii) NVT simulation at *T* = 10 K with restraints on solute heavy atoms for 12 ps, (iii) NPT MD simulation at *T* = 10 K with restraints on solute heavy atoms for 12 ps, (iv) NPT MD simulation at *T* = 300 K with restraints on solute heavy atoms for 12 ps, and (v) NPT MD simulation at *T* = 300 K without restraints for 24 ps. Subsequently, a series of consecutive 7 × 50 ns restrained MD simulations were performed in which the restraint force was gradually decreased from 1.0 to 0.0 kcal mol^–1^ Å^–2^ followed by 200 ns unrestrained MD run. The restraints were applied on the backbone atoms of IRE1 and mRNA XBP1. Each sequential MD simulation was started from the last snapshot of the preceding simulation. From the unrestrained MD simulation, one snapshot was chosen to be used as a starting input structure in the QM/MM WT-MetaD simulation.

### QM/MM WT-MetaD

The CP2K code^32,33^ version 8.2, patched with the enhanced sampling library PLUMED 2.7.2,^34,35^ was employed to perform QM/MM WT-MetaD. The system was solvated in a cubic box with TIP3P^24^ water molecules and 10 Å buffer distance. The system was neutralized by adding Na^+^ ions, and a 0.15 M MgCl_2_ salt concentration was implemented. The QM subsystem (Figure 2C) includes the residues C33, G34, C35, and A36 of mRNA XBP1 and side chain atoms of the residues within 6.0 Å distance from the scissile phosphate: Y982, H910, R905, N906, and H909. Residues C33 and A36 were cut through the C–C bonds in 5′ and 3′ positions, respectively. The side chains of amino acid residues were cut through the C_α_–C_β_ bonds such that the C_α_ and C_β_ atoms were part of the MM and QM subsystems, respectively. Linker atoms between QM and MM subsystems were treated using the integrated molecular orbital molecular mechanics (IMOMM) method.^36^ The rest of the system was considered as part of the MM subsystem. The semiempirical parametrization method 6 (PM6)^37^ level of theory along with the Grimme dispersion interaction correction (PM6-D3)^38^ were applied to the QM subsystem. It has been shown that incorporation of the dispersion interaction correction term into the PM6 potential (PM6-D3) improves protein–ligand interaction energies.^39^ The PM6 level of theory has been extensively evaluated for biological systems^40−42^ and material science^43−45^ and machine learning based studies^46,47^ and provides a very good trade-off between the speed of force field techniques and the accuracy of *ab initio* approaches. Therefore, it allows extensive sampling of large (bio)systems, while enabling one to consider the impact of electronic structure changes.^48^ In addition, it has recently been proven^49,50^ that the performance of PM6 is comparable with the very accurate second-order Møller–Plesset perturbation theory (MP2) approach.^51^ Moreover, Chen et al.^52^ showed that the accuracy of intermolecular interactions in biological systems (hydrogen bonding and polar interactions) using QM/MM MD simulation with PM6 can reach that obtained in density functional theory (DFT)-based QM/MM MD simulation. Stewart^40^ verified the applicability of the PM6 method in protein modeling and evaluation of the biocatalytic reaction in the chymotrypsin-catalyzed hydrolysis of a peptide bond. PM6-D3 level of theory has furthermore been recommended as a suitable technique for large host–guest systems.^53^

The MM subsystem was modeled using the side chain and backbone-modified AMBER14 force field (ff14SB)^54^ along with the OL3 parameters for RNA molecules.^55^ Prior to the QM/MM-WT-MetaD simulation, the system was subjected to 1000 steps of energy minimization using the limited-memory Broyden–Fletcher–Goldfarb–Shanno (LBFGS) algorithm^56^ and equilibrated by conducting 1 ns NVT followed by 2 ns NPT classical MD simulations, where the temperature and pressure were controlled at 298 K and 1 atm using canonical sampling through a velocity rescaling (CSVR)^57^ thermostat and barostat, respectively, with a 100 fs relaxation time in both and a fixed time step of 0.5 fs. A 10 Å cutoff distance was applied for nonbonded interactions while the smooth particle-mesh Ewald (SPME) technique^30^ was used for the long-range electrostatic interactions.

The collective variables (CVs) for the QM/MM WT-MetaD simulation were carefully chosen to fully represent the RNA cleavage reaction (Figure 2C): CV1 represents the nucleophilic attack of O2′ to the phosphorus atom of the scissile phosphate (SP) that results in formation of the O2′–SP (d1) and breakage of the O5′–SP (d2) bonds. Therefore, CV1 was thus considered as the length difference between d1 and d2 (CV1 = d1 – d2). CV2 represents the activation of the nucleophile atom O2′ through a proton transfer reaction from O2′ atom to Nε atom of GB(H910) which leads to breakage of O2′–H (d3) and formation of Nε–H (d4) bonds (CV2 = d3 – d4). CV3 represents the proton transfer reaction from the side chain hydroxyl group of GA(Y892) to the recently detached cytidine O5′ atom which results in breakage of O–H (d5) and formation of O5′–H (d6) bonds (CV3 = d5 – d6). To enhance the sampling procedure of the CV space, the system was biased by adding a Gaussian kernel with the initial height of 0.5 kcal mol^–1^ and width of 0.1 Å every 100 MD steps (50 fs). A biasing factor of 17.89 (i.e., 10 kcal mol^–1^) was set to scale down the heights of spawning Gaussian kernels. The rest of the settings were the same as in classical MD. The QM/MM WT-MetaD simulations stopped when the convergence criteria were satisfied. The reweighting (unbiasing) method and block analysis were used to monitor the convergence and to calculate the errors in the free energy estimation.

## Results and Discussion

### Construction of the XBP1 mRNA Single Stem-Loop

Figure 2A and B shows 2D and 3D structural comparisons between the template tRNA^Phe^ and mRNA XBP1 single stem-loops. As Figure 2A indicates, the conserved sequence of “CNGNNGN”, which is a fingerprint characterization of IRE1 substrates, exists within the stem-loop of both structures. The cleavage sites in tRNA^Phe^ and XBP1 are GC and GA, respectively (Figure 2A). The dissimilarity in nucleobase composition after the mutations causes some variations in the atomic coordinates, especially in the case of purine to pyrimidine mutations. The possible close atomic contacts and clashes were resolved by system preparation followed by restrained minimization. Moreover, the final IRE1/XBP1 mRNA complex was subjected to multiple restrained MD refinement steps which further improved the quality of the XBP1 mRNA stem-loop structure and the interaction network in the interface of the protein and RNA. The template-based approach used herein was preferred over *de novo* RNA modeling of XBP1 mRNA because of its 3D complexity, which is challenging for RNA structure prediction engines.^58^ As Figure 2D illustrates, the final unconstrained MD simulation shows that the XBP1 mRNA model remained stable during the simulation.

### Molecular Docking

**Figure 3:**
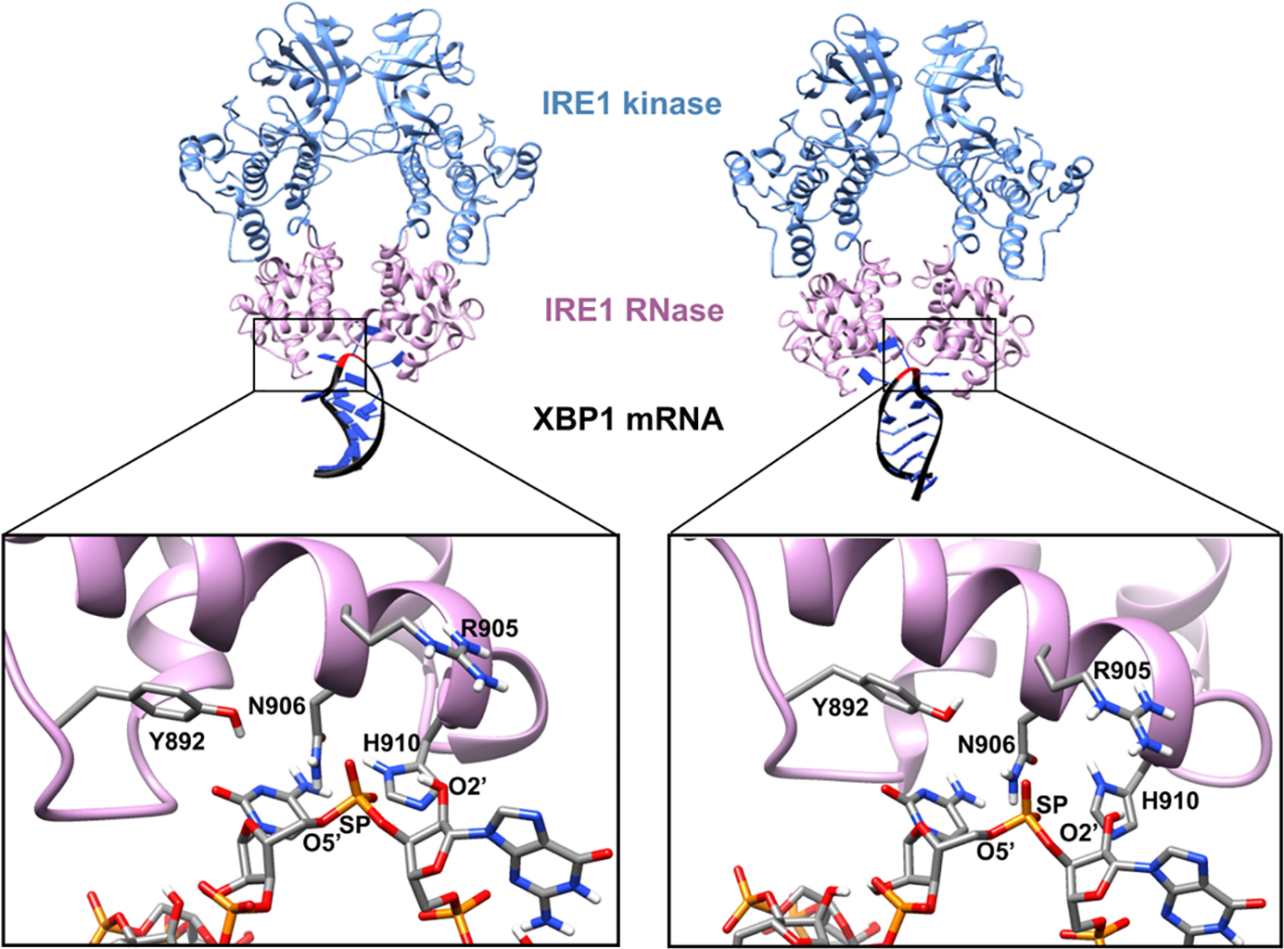
Best docking poses (Model_A1 and Model_B1) generated by molecular docking. The top panel shows the entire complexes where the Kinase and RNase domains of the IRE1 dimer are shown in blue and pink, respectively. The XBP1 mRNA single stem-loop is represented as ribbon (black) and tubes/slabs (dark blue). The cleavage site of the XBP1 mRNA is highlighted in red. The bottom panel is a zoomed-in illustration of the active sites in the RNase domain of each IRE1 monomer along with the residues important in the cleavage mechanism discussed in the text. The atoms in the active site are colored as carbon in gray, hydrogen in white, nitrogen in blue, oxygen in red, and phosphorus in orange. Hydrogen atoms bound to carbon are omitted for clarity. The luminal and transmembrane domains of *h*IRE1 are not shown in the figure.

Constrained Protein–RNA docking was employed to generate IRE1 back-to-back dimer/XBP1 mRNA single stem-loop complexes (Figure 3). Molecular docking resulted in 20 poses (10 poses toward each catalytic center). Table 1 shows the docking score values associated with the docking poses. As Table 1 indicates, the best models (Model_A1 and Model_B1) have significantly better docking scores compared to the other models. Model_A1 and Model_B1 not only have similar docking scores but the XBP1 mRNAs stem-loops are also bound in an equivalent orientation into the RNase cleft of the *h*IRE1 dimer, where the scissile phosphate groups were favorably positioned for a GA–GB mechanism. The best two docking models (Model_A1 and Model_B1) are shown in Figure 3. A closer inspection revealed the following: (a) H910 is positioned to act as a GB by abstracting proton from the O2′ atom of guanosine and activate the nucleophile. (b) Y892 is well oriented to act as a GA by donating its proton to the cytidine O5′ atom. (c) R905 and N906 are positioned to alleviate the negative charge on the scissile phosphate and hold it in a position suitable for the reaction. (d) The O2′–SP–O5′ angle is rather close to linear (∼ −150° for both monomers) which is believed to be relevant for a “S_N_2-like” nucleophile attack reaction mechanism.^59^ These results confirm the successful molecular docking calculations.

**Table 1:** Docking Score Values Associated with Each Docking Model Obtained from the HDOCK Docking Engine

Toward catalytic center of monomer A	Docking score	Toward catalytic center of monomer B	Docking score
Model_A1	–293.82	Model_B1	–294.33
Model_A2	–268.61	Model_B2	–264.58
Model_A3	–262.03	Model_B3	–254.40
Model_A4	–249.81	Model_B4	–254.11
Model_A5	–249.60	Model_B5	–249.97
Model_A6	–242.08	Model_B6	–249.13
Model_A7	–232.17	Model_B7	–241.60
Model_A8	–230.52	Model_B8	–238.77
Model_A9	–228.99	Model_B9	–231.27
Model_A10	–227.61	Model_B10	–226.96

### Molecular Dynamics Simulations

**Figure 4:**
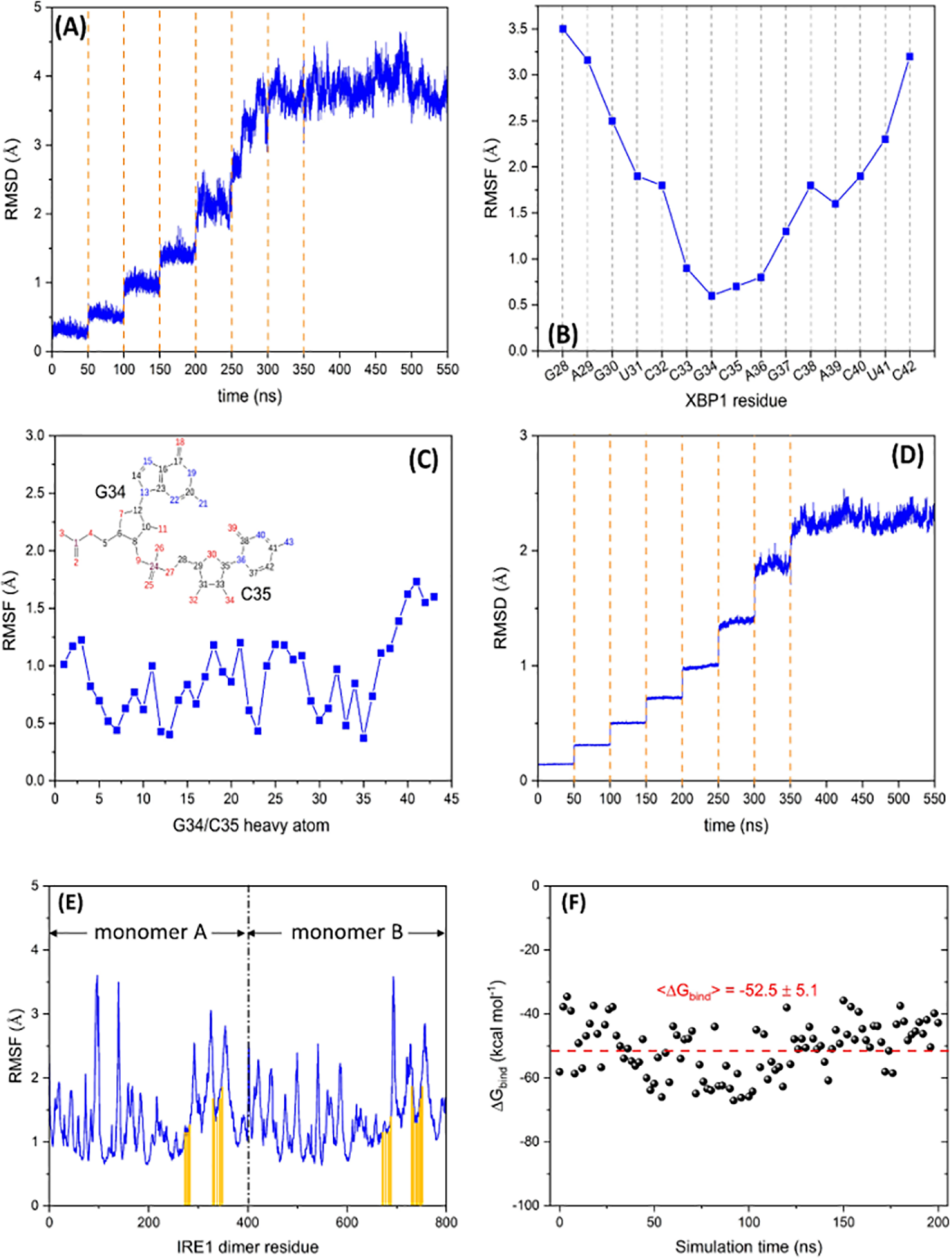
Backbone (A) RMSD and (B) RMSF of the XBP1 mRNA bound to the *h*IRE1 back-to-back dimer during a consecutive series of 7 × 50 ns restrained MD simulations followed by a final 200 ns unrestrained run. (C) Heavy-atom RMSF of the XBP1 mRNA binding nucleotides (G34/C35) during the classical MD simulation. Backbone (D) RMSD and (E) RMSF of the *h*IRE1 dimer bound to XBP1 mRNA during the MD simulations. The interacting residues of the *h*IRE1 dimer with XBP1 mRNA are marked with orange vertical bars. (F) Free energy of binding (Δ*G*_bind_) between the *h*IRE1 dimer and XBP1 mRNA calculated for 100 snapshots extracted from the 200 ns unrestrained MD trajectory.

**Figure 5:**
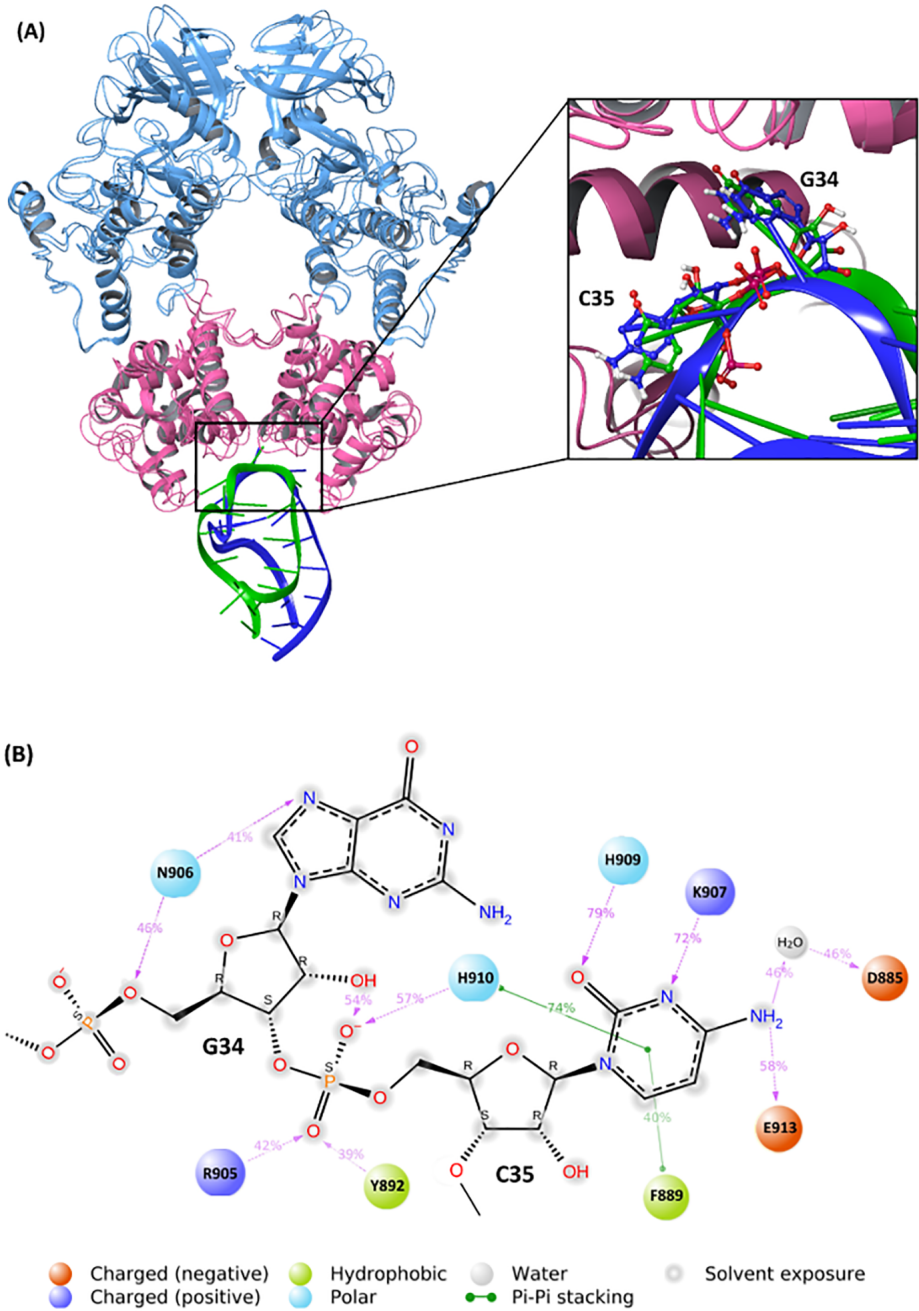
(A) Last snapshot of the MD simulation (*t* = 550 ns) superposed on the initial structure. The XBP1 mRNA in the first and last snapshots are presented in dark blue and green colors, respectively. The zoomed-in picture illustrates the nucleotides G34 and C35 bound to the RNA binding site. The Kinase and RNase domains of the *h*IRE1 dimer are shown in light blue and pink, respectively. (B) Abundance of atomic interactions between the *h*IRE1 dimer and nucleotides G34/C35 of XBP1 mRNA evaluated during the 200 ns unrestrained MD simulation.

Since Model_A1 and Model_B1 are equivalent (Table 1 and Figure 3), one of the models (Model_A1) was selected and subjected to multiple restrained MD simulation refinement to better sample the conformational space. The IRE1–XBP1 mRNA interface is sequence and structure specific,^9,10^ and the protein–RNA interface contains a complex network of interactions. These sort of convoluted protein–RNA complexes have shown to be challenging to be accurately described using traditional MD simulations.^60,61^ Therefore, restraints^62^ were applied during MD simulations to gradually relax the complex from the initially docked conformation and allow sampling of different conformational states without disrupting too much the interactions at the protein–RNA interface. Figure 4A shows the backbone RMSD of XBP1 mRNA bound to the RNase domain of the IRE1 dimer during a series of 7 × 50 ns consecutive restrained MD simulations followed by a final 200 ns unrestrained run. As Figure 4A indicates, the backbone RMSD of XBP1 mRNA reaches a converged value of ∼4 Å. This is relatively high and implies some conformational changes during the MD simulation. To further evaluate this conformational change, the backbone root mean squared fluctuation (RMSF) of XBP1 mRNA was calculated and is presented in Figure 4B. As Figures 4B and 5A show, the main conformational changes occur at the two free ends of XBP1 mRNA while the binding region (G34 and C35) remains almost intact (RMSF = 0.7–0.8 Å) when compared to the initial structure (Model_A1). Figure 4C illustrates the heavy-atom RMSF graph of G34 and C35 with an average value of 0.9 Å, confirming the minor movement of the binding nucleotides. The dynamical behavior and possible conformational reorientation of the *h*IRE1 dimer as a receptor protein was also investigated through the backbone RMSD and RMSF graphs, shown in the Figure 4D and E, respectively. Figure 4D indicates that the backbone RMSD of the dimer IRE1 reaches a plateau at ∼2.3 Å while the average backbone RMSF of the interacting residues in the RNA binding site (marked with orange vertical bars in Figure 4E) is ∼1.5 Å.

Figure 5A shows the last snapshot of the MD simulation (*t* = 550 ns) superposed on the initial structure, and the zoomed-in picture illustrates the nucleotides G34 and C35 bound to the RNA binding site. The abundance of atomic interactions between the *h*IRE1 dimer and nucleotides G34/C35 of XBP1 mRNA was evaluated during the last 200 ns unrestrained MD simulation (Figure 5B). The catalytic residue H910 (GB) forms a hydrogen bonding interaction with a nonbridging oxygen atom of the scissile phosphate group (close to the nucleophile O2′ atom) along with a π–π stacking with the pyrimidine ring of C35. These interactions are strong enough to keep H910 in the vicinity of the nucleophile O2′ atom. Y892 (GA) and R905 form hydrogen bonding interactions with the other nonbridging oxygen atom of the scissile phosphate (close to the leaving group O5′ atom). N906 forms two hydrogen bonds with a nitrogen atom of the purine ring and the O5′ atom of G34, keeping this nucleotide fixed. The free energy of binding between *h*IRE1 dimer and XBP1 mRNA (Δ*G*_bind_) has been estimated using molecular mechanics with generalized Born surface area (MM/GBSA)^63^ as implemented in the Schrodinger package. The average free energy of binding ⟨Δ*G*_bind_⟩ was calculated over 100 snapshots extracted from 200 ns unrestrained MD simulation (every 2 ns) and is presented in Figure 4F. The negative average value (−52.5 ± 5.1 kcal mol^–1^) confirms that the interaction between the protein and RNA is favorable, and the resulting complex is stable.

From the second half of the unrestrained MD simulation where the complex is fully relaxed and equilibrated, a plausible conformation was identified to be used as an initial structure in the subsequent QM/MM WT-MetaD simulation. A conformation was chosen in which the d4 and d6 distances (Figure 2C) were simultaneously below 5 Å. Table 2 lists the atomic distances of the selected structure from the MD simulation trajectory.

**Table 2:** Interatomic Distances and CV Values of the Selected MD Snapshot and Stationary Points on the Free Energy Surface Obtained from the QM/MM WT-MetaD Simulation.^a^

Structure	d1	d2	d3	d4	d5	d6	CV1	CV2	CV3
MD snapshot	2.21	1.68	1.02	4.57	1.01	4.48	–	–	–
A	2.23	1.66	1.00	2.88	1.05	2.91	0.57	–1.88	–1.86
A′	2.09	1.70	1.15	1.33	1.04	3.04	0.39	–0.18	–2.00
B	1.77	1.72	2.99	1.03	1.05	3.03	0.05	1.96	–1.98
B′	1.65	1.78	2.98	1.03	1.22	1.49	–0.13	1.95	–0.27
C	1.66	2.17	2.99	1.04	2.98	1.03	–0.51	1.95	1.95
C′	1.65	2.74	2.98	1.03	2.96	1.04	–1.10	1.95	1.92
D	1.66	5.51	3.05	1.03	3.03	1.03	–3.85	2.02	2.00
D′	1.66	5.57	3.01	1.05	1.16	1.31	–3.91	1.96	–0.15
E	1.66	5.37	3.01	1.04	1.05	3.06	–3.71	1.97	–2.01
B″	1.67	2.64	3.02	1.03	1.04	3.00	–0.97	1.99	–1.96

a Interatomic distances d1–d6 refer to Figure 2C.

### QM/MM WT-MetaD

**Figure 6:**
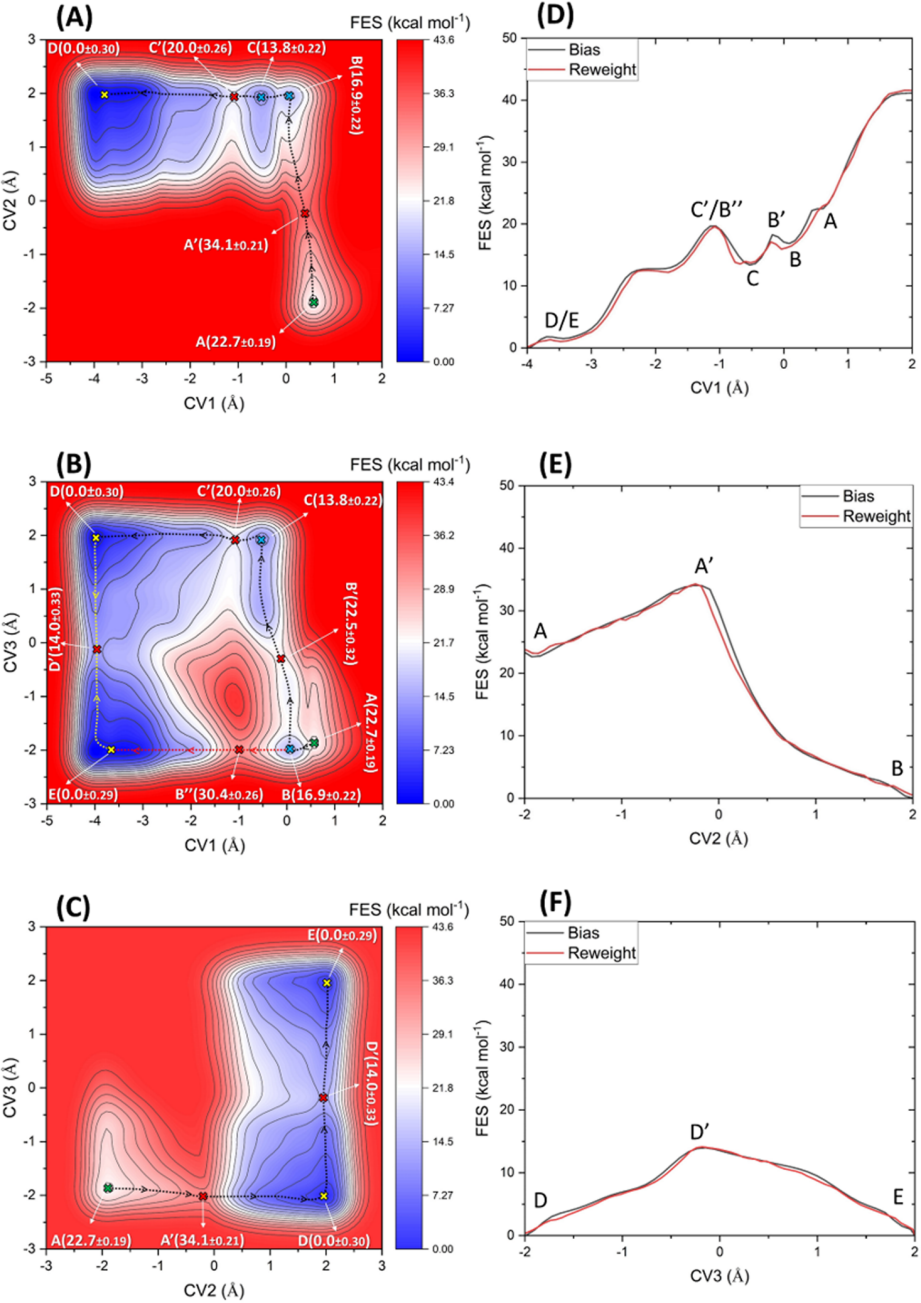
2D projected free energy surface (FES) contour map of (A) CV1/CV2, (B) CV1/CV3, and (C) CV2/CV3. In each panel, the CV coordinates of the reactant structure (after classical minimization and equilibration steps), products, local minima, and saddle points are highlighted by green, yellow, blue, and red crosses, respectively. The main and alternative reaction paths are shown by black and red dashed lines, respectively. The numbers within the parentheses indicate the relative free energy values at each point. 1D projected free energy profiles of (D) CV1, (E) CV2, and (F) CV3, calculated from the negative of the cumulative biasing potential (black line) compared with those obtained by the reweighting technique (red line).

Figure 6A–C shows the 2D projected free energy surface (FES) contour maps for CV1/CV2, CV1/CV3, and CV2/CV3, respectively. The CV coordinates of the reactant structure (after minimization and equilibration steps), products, local minima, and saddle points are highlighted by green, yellow, blue, and red crosses, respectively. The minimum free energy paths (MEP) are shown as dashed lines. As Figure 6 shows, the initial structure, point A, was located at a local minimum with CV1 = 0.57 Å, CV2 = −1.88 Å, and CV3 = −1.86 Å, hereafter labeled as A (0.57, −1.88, −1.86). Moving along the MEP from points A (0.57, −1.88, −1.86) to B (0.05, 1.96, −1.98), Figure 6A corresponds to the proton transfer from the nucleophile O2′ atom to the Nε atom of GB H910. This activates the nucleophilic attack on the SP atom, leading to the formation of a pentavalent dianionic phosphate intermediate (point B). This reaction requires passing through a transition state, point A′ (0.39, −0.18, −2.00), with an activation energy of 11.4 ± 0.21 kcal mol^–1^. As Table 2 shows, the interatomic distance d1 decreases from 2.23 Å in point A to 2.09 Å in point A′ and 1.77 Å in point B, while d2 increases from 1.66 Å → 1.70 Å → 1.72 Å in points A, A′, and B, respectively. The transition state A′ is thus located intermediately along the proton transfer and nucleophile activation pathway. For d3 and d4, which are involved in this pathway, the transition structure A′ lies very early for the breakage of the O2′–H bond where d3 increases from 1.00 Å (point A) to 1.15 (point A′) and 2.99 (point B), while for d4, i.e., the protonation of Nε, A′ is a very late TS in that d4 decreases from 2.88 to 1.33 Å to 1.03 Å in points A, A′, and B, respectively.

Moving along the MEP (black dashed line) from points B (0.05, 1.96, −1.98) to C (−0.51, 1.95, 1.95) involves proton transfer from the hydroxyl group of GA Y892 to the ready-to-leave O5′ atom. This proton transfer reaction passes through a transition state, point B′ (−0.13, 1.95, −0.27) with an activation energy of 5.6 ± 0.32 kcal mol^–1^. The corresponding distance d5 increases from 1.05 Å (point B) to 1.22 Å (point B′) to 2.98 Å (point C), indicative of an early transition state. At the acceptor end, d6 (H–O5′) decreases from 3.03 Å → 1.49 Å → 1.03 Å in points B, B′, and C, respectively. Concomitant with this proton transfer, the pentavalent phosphate intermediate structure breaks, giving the O2′–SP–O3′ cyclic intermediate. The O2′–SP interatomic distance d1 thereby decreases from 1.77 Å in point B and 1.65 Å in point B′ and 1.66 Å in point C, while d2 (SP–O5′) increases from 1.72 to 1.78 Å to 2.17 Å in points B, B′, and C, respectively. While the O5′ atom in point C is fully protonated, it still has a weak interaction with the SP atom. The protonated O5′ atom in point C decouples from the dianionic phosphate group by passing through a transition state C′ (−1.1, 1.95, 1.92) (Figure 4A) with an activation energy of 6.2 ± 0.26 kcal mol^–1^ to reach one of the products, point D (−3.85, 2.02, 2.00), where the GA is deprotonated. This reaction is associated with the elongation of the interatomic distance d2 from 2.17 Å → 2.74 Å → 5.51 Å in points C, C′, and D, respectively.

**Figure 7:**
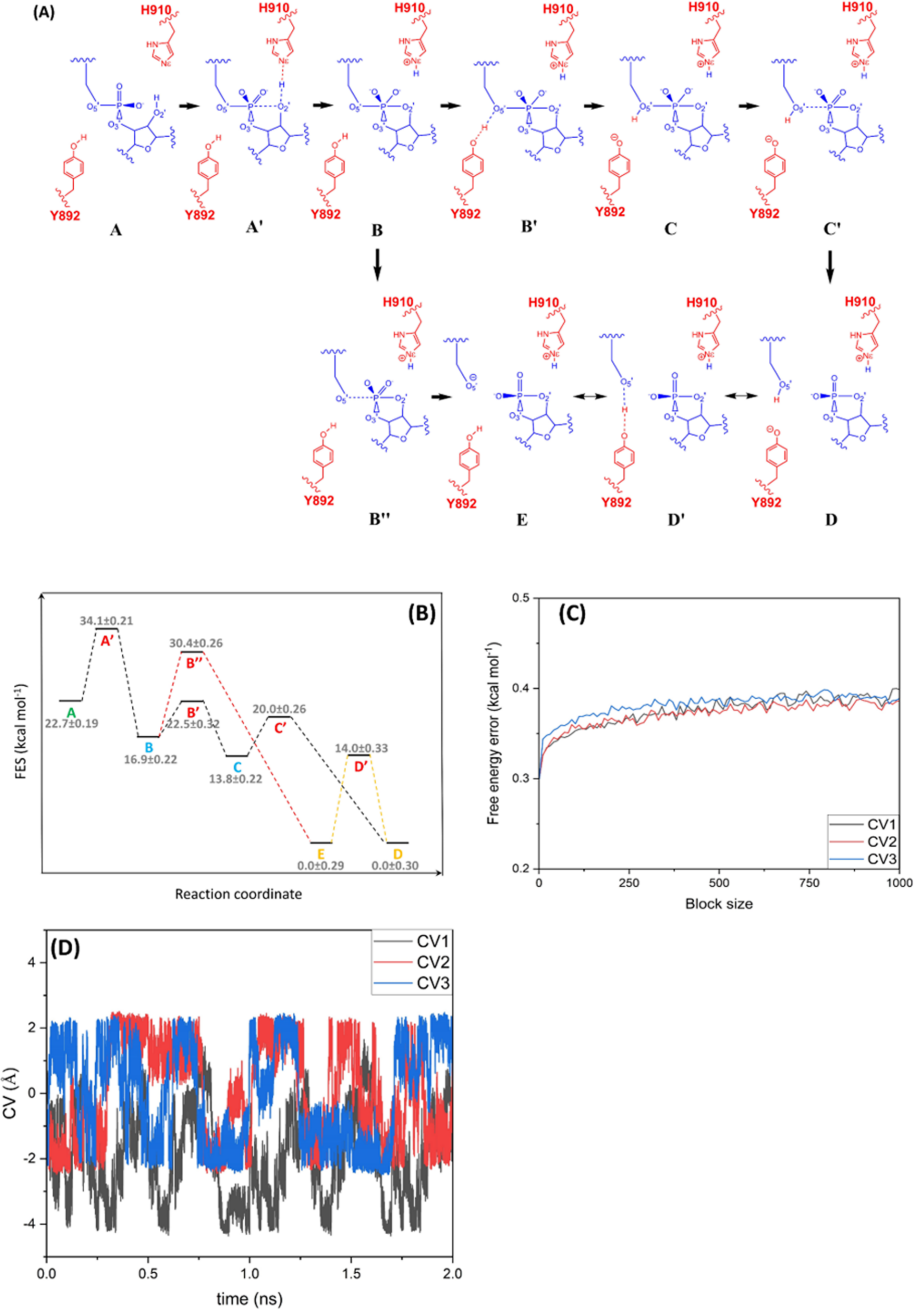
(A) Unconventional cleavage mechanism of mRNA XBP1 by IRE1 elucidated from QM/MM WT-MetaD simulations. The IRE1 and XBP1 atoms involved in the catalytic reaction are shown in red and blue, respectively. (B) Relative free energy diagram of the reactant, products, intermediates, and transition states. (C) Error in free energy estimation for each CV calculated by the block-average technique. (D) Diffusive behavior of the CV sampling during the simulation trajectory.

As Figures 6B and 7A illustrate, there is an alternative reaction path (red dashed line) where the XBP1 mRNA cleavage proceeds without direct contribution of GA in the proton transfer to the leaving O5′ atom. This alternative starts from the local minimum point B (0.05, 1.96, −1.98), passes through a transition state, point B″ (−0.97, 1.99, −1.96), with activation energy 13.5 ± 0.26 kcal mol^–1^, and reaches the other product, point E (−3.71, 1.97, −2.00). During this process, the interatomic distance d1 (O2′–SP) decreases from 1.77 Å in point B to 1.67 Å in point B″ and 1.66 Å in point E, while the SP–O5′ distance d2 increases from 1.72 to 2.64 Å to 5.37 Å in points B, B″, and E, respectively. The main reaction path (black dashed line in Figure 4B) is more favorable compared to the alternative one since the first activation energy of the main reaction path (B to B′, 5.6 ± 0.32 kcal mol^–1^) is significantly lower than that of the alternative path (B to B″, 13.5 ± 0.26 kcal mol^–1^) (Figure 5B). The end-point products from each reaction path (points E and D) are connected through a proton transfer reaction between Y892 and O5′ which requires passing through a transition state, point D′ (−3.91, 1.96, −0.15), with an activation energy of 14.0 ± 0.33 kcal mol^–1^. The main indicators for this conversion reaction are interatomic distances d5 and d6, where d5 decreases from 3.03 Å in point D to 1.16 Å in point D′ and 1.05 Å in point E, while d6 increases from 1.03 to 1.31 Å to 3.06 Å in points D, D′, and E, respectively.

The results clearly show that the first stage of the XBP1 cleavage reaction (points A to B), i.e., activation of the nucleophile O2′ atom after proton transfer to the GB, nucleophilic attack to the SP atom, and formation of the dianionic pentavalent phosphate, is an essential step in both main and alternative reaction paths. From point B, the reaction can proceed through two different paths as outlined above. In the main reaction path with a lower activation energy (black dashed line in Figure 4B), Y892 as GA directly contributes to the reaction by donating the proton to the leaving O5′ atom, while in the alternative path (red dashed line), Y892 does not contribute directly to the cleavage reaction and stays protonated (Figure 5A). As Figure 5B indicates, the activation energy of the main reaction path is significantly lower than the alternative pathway, in agreement with the experimental observation that GB mutation of the *y*IRE1 RNase reduces the reaction rate by >300,000-fold while the GA mutation only reduces the reaction rate by ∼10-fold.^4^

To the best of our knowledge, this work is the first *in silico* study ascertaining that a Tyrosine residue can be considered as GA in the catalytic reaction mechanism of an RNase enzyme, which successfully confirms the experimental IRE1 mutagenesis data.^4^ However, the contribution of a Tyrosine residue as a potential proton-donor entity (GA) has previously been suggested experimentally in the catalytic activity of HigB toxin RNase.^64^ The p*K*_a_ value of a solvent-exposed tyrosine residue is typically ∼10, and therefore, to contribute as a GA at neutral pH, the p*K*_a_ would need to be perturbed. One possible explanation is that the local microenvironment of the IRE1 dimer bound to the XBP1 mRNA alters the p*K*_a_ of Tyr892, making it more suitable to function as a proton donor. Evidence for this includes the protein Ketosteroid isomerase where the p*K*_a_ of an active site tyrosine residue is perturbed to ∼6.^65^ Another possibility is based on the fact that the p*K*_a_ of the oxyanion leaving group (O5′ atom) is much higher than the tyrosine side chain. The accumulated negative charge on the oxyanion leaving group could promote proton donation by Tyr892.^64^

The reweighting technique of Bonomi et al.^66^ was applied to the simulation trajectory as a convergence test and error estimation method by comparing the free energy profiles of each CV obtained from the negative of the cumulative biasing potential with those evaluated by the reweighting technique. The results are shown in Figure 6D–F. As shown, the 1D free energy profiles of the CVs obtained from the two approaches are very consistent. The average unsigned errors between the points are 0.36, 0.37, and 0.35 kcal mol^–1^ for CV1, CV2, and CV3, respectively, which can be related to the finite-length simulation.^67^ Moreover, block-average analysis^68,69^ was used to further assess the convergence of the simulations and the associated statistical errors. In the block-average analysis technique, the simulation trajectory is divided into a set of blocks with equal lengths. The error in the free energy estimation can be calculated by comparing the average free energy values from each block. When the number of blocks is large enough, the average error should not be time correlated. Figure 7C illustrates the block-average analysis results for three CVs with 1000 blocks. As Figure 7C shows, the errors in free energy estimation for all three CVs are perfectly converged to a value less than 0.4 kcal mol^–1^ which is in agreement with the results obtained from the reweighting technique mentioned above. The results from the reweighting scheme (Figure 6D–F) along with those from block-average analysis technique (Figure 7C) and the diffusive behavior of the CV sampling (Figure 7D) clearly confirm that the QM/MM WT-MetaD simulation has fully converged after 2 ns.

### Previous Studies on Relevant Systems

RNase enzymes can generally be categorized into two main classes:^70^ metal ion dependent and independent. Metal ion-independent RNase enzymes (like IRE1) produce RNA fragments with 2′,3′-cyclic phosphates, whereas metal ion-dependent enzymes do not. While the catalytic reaction mechanisms of metal ion-dependent and metal ion-independent RNase are totally different, they can also differ substantially within each class. Many of these reaction mechanisms (mainly for metal ion-dependent RNases) have been evaluated and confirmed by *in silico* methods. In this section, we review previous studies on similar systems leading to catalytic cleavage of RNA molecules by RNase enzymes and compare the results.

Mlynsky et al.^71^ conducted a benchmarking study on the performances of different computational techniques, including post Hartree–Fock *ab initio* (MP2), DFT (BLYP and MPW1K), and semiempirical (AM1 and DFTBPR), in determining the catalytic mechanism of hairpin ribozyme cleavage in the context of a GA–GB reaction. The main aspect of their work was to generate the potential energy surface (PES) of the reaction by scanning the relevant bonds breaking and forming during the cleavage reaction using different computational techniques. The PES profiles indicated that MP2 and DFT methods estimate a maximum activation energy of 10–18 kcal mol^–1^ which is in agreement with the maximum activation energies calculated in this study (11–14 kcal mol^–1^). On the other hand, semiempirical techniques yielded a relatively high activation energy (∼32 kcal mol^–1^) on the PES profile. However, the authors also conducted a QM/MM MD simulation at the semiempirical AM1 level of theory and umbrella sampling to generate a free energy surface (FES) profile instead of PES, which resulted in a more reasonable activation energy (18 kcal mol^–1^) consistent with the data obtained by the MP2 and DFT techniques. It shows that an FES profile from QM/MM MD simulations is more accurate and reliable than a PES profile from a simple scanning calculations, at least for semiempirical techniques. It is worth mentioning that MP2 and DFT QM/MM MD simulations were not evaluated because of the very demanding computational requirements.

Elsässer et al.^72^ evaluated the RNA cleavage mechanism of metal ion-independent enzyme “RNase A” by means of QM/MM techniques (B3LYP/AMBER99). Their results showed a low energy barrier (7.5–10 kcal mol^–1^) in which one His residue facilitates the activation of the nucleophile while another His residue acts as GA and protonates the leaving group. The catalytic mechanism of RNA cleavage by “RNase H” has been studied by Rosta et al.^73^ using QM/MM with DFT (B3LYP) and the CHARMM force field. Based on their findings, a deprotonated water molecule mediated by phosphate/Mg^2+^ acts as the nucleophile while a protonated Asp residue protonates the leaving group. The overall reaction barrier was estimated to 15 kcal mol^–1^. Casalino et al.^74^ employed DFT (B3LYP/BLYP) and the Amber ff12SB force field to conduct a series of QM/MM MD simulations in combination with thermodynamic integration to reveal the atomistic details behind the cleavage mechanism of Group II introns ribozymes. They showed that the energy barrier of the rate-determining step is 18.8 kcal mol^–1^, in line with the experimental catalytic rate. Casalino et al.^75^ evaluated the catalytic mechanism of nontarget DNA cleavage using QM/MM MD simulations with DFT (BLYP) and the Amber ff12SB force field. They obtained an activation barrier energy of 15.5 kcal mol^–1^ for the rate-determining step, in agreement with experimental data. Using DFT (B3LYP) and CHARMM 27 force field, Dürr et al.^76^ conducted a series of extensive QM/MM calculations combined with Hamiltonian replica exchange to elucidate the RNA cleavage mechanism of HIV-1 “RNase H”. They found an overall reaction barrier of ∼19 kcal mol^–1^, associated with the phosphate-cleavage step, which matches the experimental rate. Drusin et al.^77^ studied the cleavage mechanism of dsRNA by bacterial “RNase III” through QM/MM MD simulations using semiempirical DFTB and the AMBER14 force field. They concluded that the energy barrier associated with this cleavage mechanism is ∼15 kcal mol^–1^. Borišek and Magistrato^78^ employed a QM/MM MD simulation (BLYP/AMBER-ff14SB) in the context of the blue moon ensemble method to study RNA catalysis in the exon-ligation step of the Spliceosome. Their results unveiled that the catalytic reaction occurs via an associative two-Mg^2+^ ion mechanism in which the scissile phosphate mediates a proton transfer from the nucleophile to the leaving group with a fee energy barrier of 14.9 kcal mol^–1^.

## Conclusions

By combining multiscale *in silico* approaches (i.e., RNA modeling, molecular docking, molecular dynamics simulations, and quantum mechanics/molecular mechanics well-tempered metadynamics simulations), the atomic details of the mechanism of XBP1 mRNA cleavage by the IRE1 RNase domain dimer were evaluated. The results show that the cleavage reaction occurs via (a) proton transfer from O2′ to the Nε atom of H910 (GB) that activates the nucleophilic attack to the scissile phosphate atom, leading to the formation of the pentavalent dianionic phosphate intermediate, (b) subsequent proton transfer from the hydroxyl group of Y892 (GA) to the ready-to-leave O5′ atom, and (c) formation of the product. In agreement with experimental evidence, an alternative reaction path with significantly higher activation energy can also take place (i.e., 13.5 kcal mol^-1^ compared to 5.6 kcal mol^–1^ for the main reaction path), in which XBP1 mRNA cleavage proceeds without direct contribution of the GA in the proton transfer to the leaving O5′ atom. This explains why mutation of the GA does not block the IRE1 RNase activity but reduces the reaction rate by ∼10-fold. In addition, the state-of-the-art molecular simulations employed in this study clearly explain the effect of the GB in driving the RNA cleavage reaction by forming hydrogen bonds with O2′ and its capability of proton acceptance from the O2′ atom. H910 is shown to be a key actor of the RNA cleavage, rationalizing the lack of IRE1 activity in the presence of GB mutation. The findings of the current study provide further advances in our understanding of the cleavage mechanism of XBP1 mRNA by IRE1 RNase and shed further light on UPR signaling.

## Data Availability

The structures of the protein–RNA docked complexes, MD trajectory files, QM/MM WT-MetaD trajectory, and a high resolution video showing how the XBP1 mRNA cleavage takes place during QM/MM WT-MetaD simulation are provided as tarballs (.tar.gz) freely accessible at https://zenodo.org/record/6457767 (DOI: 10.5281/zenodo.6457767).

## Supporting Information Available

Video showing how the XBP1 mRNA cleavage takes place during the QM/MM WT-MetaD simulation (MPG)

## Supplementary Material

ci2c00735_si_001.mpg
